# MAGnesium sulphate for fetal neuroprotection to prevent Cerebral Palsy (MAG-CP)—implementation of a national guideline in Canada

**DOI:** 10.1186/s13012-017-0702-9

**Published:** 2018-01-11

**Authors:** Dane A. De Silva, Anne R. Synnes, Peter von Dadelszen, Tang Lee, Jeffrey N. Bone, MAG-CP, CPN and CNN collaborative groups, Laura A. Magee

**Affiliations:** 1 Department of Obstetrics and Gynaecology, University of British Columbia, Vancouver, Canada; 2 BC Children’s Hospital Research Institute, University of British Columbia, Vancouver, Canada; 3 Department of Paediatrics, University of British Columbia, Vancouver, Canada; 4 Department of Women and Children’s Health, St Thomas’ Hospital, 10th Floor, North Wing, Westminster Bridge Road, London, SE1 7EH UK; 5 School of Life Course Sciences, Faculty of Life Sciences and Medicine, King’s College London, London, UK

**Keywords:** Preterm birth, Fetal neuroprotection, Magnesium sulphate, Cerebral palsy, Knowledge translation, Implementation, Interrupted time-series

## Abstract

**Background:**

Evidence supports magnesium sulphate (MgSO4) for women at risk of imminent birth at < 32–34 weeks to reduce the likelihood of cerebral palsy in the child. MAGnesium sulphate for fetal neuroprotection to prevent Cerebral Palsy (MAG-CP) was a multifaceted knowledge translation (KT) strategy for this practice.

**Methods:**

The KT strategy included national clinical practice guidelines, a national online e-learning module and, at MAG-CP sites, educational rounds, focus group discussions and surveys of barriers and facilitators. Participating sites contributed data on pregnancies with threatened very preterm birth. In an interrupted time-series study design, MgSO4 use for fetal neuroprotection (NP) was tracked prior to (Aug 2005–May 2011) and during (Jun 2011–Sept 2015) the KT intervention. Effectiveness of the strategy was measured by optimal MgSO4 use (i.e. administration when and only when indicated) over time, evaluated by a segmented generalised estimating equations logistic regression (*p* < 0.05 significant). Secondary outcomes included maternal effects and, using the Canadian Neonatal Network (CNN) database, national trends in MgSO4 use for fetal NP and associated neonatal resuscitation. With an anticipated recruitment of 3752 mothers over 4 years at Canadian Perinatal Network sites, we anticipated > 95% power to detect an increase in optimal MgSO4 use for fetal NP from < 5 to 80% (2-sided, alpha 0.05) and at least 80% power to detect any increases observed in maternal side effects from RCTs.

**Results:**

Seven thousand eight hundred eighty-eight women with imminent preterm birth were eligible for MgSO4 for fetal NP: 4745 pre-KT (18 centres) and 3143 during KT (11 centres). The KT intervention was associated with an 84% increase in the odds of optimal use (OR 1.00 to 1.84, *p* < 0.001), a reduction in the odds of underuse (OR 1.00 to 0.47, *p* < 0.001) and an increase in suboptimal use (too early or at ≥ 32 weeks; OR 1.18 to 2.18, *p* < 0.001) of MgSO4 for fetal NP. Maternal hypotension was uncommon (7/1512, 0.5%). Nationally, intensive neonatal resuscitation decreased (*p* = 0.024) despite rising MgSO4 use for fetal NP (*p* < 0.001).

**Conclusion:**

Multifaceted KT was associated with significant increases in use of MgSO4 for fetal NP, with neither important maternal nor neonatal risks.

**Electronic supplementary material:**

The online version of this article (10.1186/s13012-017-0702-9) contains supplementary material, which is available to authorized users.

## Background

Complicating approximately 10% of births, prematurity remains a major cause of perinatal mortality and morbidity, especially cerebral palsy (CP) [1–3]. Although survival rates of babies born preterm have risen, there has been no parallel fall in neurodevelopmental impairment rates, especially among babies born very preterm at < 32 weeks’ gestation [4].

By 2009, meta-analyses of randomised controlled trials (published between 2002 and 2008 [5–8]) had shown that antenatal MgSO4 administered for fetal neuroprotection (NP) at < 32–34 weeks reduces the likelihood of CP (relative risk (RR) 0.68 [0.54, 0.87]) [9–11]. However, controversies remained about this therapy, including concerns about potential effects of MgSO4 on fetal heart rate [12] and increased neonatal resuscitation [13], a lack of understanding of the neuroprotective mechanism of action [14] and inadequate studies describing long-term adverse paediatric outcomes other than CP.

### Rationale

As antenatal corticosteroids prior to preterm delivery were not routinely administered in North America until 22 years after their benefit had been established, we anticipated that implementation of MgSO4 for fetal NP into clinical practice would require a knowledge translation (KT) intervention. A previous study on existing knowledge resources about MgSO4 for fetal NP in Canada found that despite convincing evidence of effectiveness, use of MgSO4 for fetal NP was near non-existent (1.5%) between 2010 and 2011, and there was no such use of MgSO4 before 2010 [15]. Still, knowledge gaps and lack of guidelines remained important barriers to use [16, 17], with the potential to cause maternal side effects an additional anticipated barrier as it has been for implementation of MgSO4 for eclampsia prevention and treatment [18]. As maternity care hospitals vary widely in terms of practices and beliefs, MAG-CP (MAGnesium sulphate for fetal neuroprotection to prevent Cerebral Palsy) was created to facilitate uptake of use of MgSO4 for fetal NP in the setting of imminent birth at < 32 weeks.

### KT strategies

We chose a multifaceted implementation strategy that was informed by the concepts of Roger’s Innovation-Diffusion theory [19], the most influential theory in knowledge utilisation [20]. This theory considers the complexity of the innovation or clinical practice, characteristics of adopters, communication channels, time considerations for adoption and uptake and organisational characteristics of the social system [19]. The process of behaviour change at the individual level includes knowledge of the innovation or clinical practice, persuasion for uptake, an individual decision for uptake and use at which point the innovation is either accepted or rejected, implementation of the innovation or clinical practice and confirmation of the decision for uptake [19]. We specifically included e-learning platforms and site outreach activities shown to support active (rather than passive) learning (especially when those activities are used in conjunction with other interventions [21, 22]) and audit and feedback that have been effective in improving practice [23–25].

### Objectives

Our primary aim was to describe our multifaceted implementation strategy and assess its effectiveness in increasing ‘optimal’ use of MgSO4 (i.e. MgSO4 administration to women delivering at under 32 weeks as indicated and no use when not indicated) to 80% of eligible women over 4 years (2011–2015), the standard benchmark for a grade 1A recommendation [26]. Our secondary objective was to report any maternal or fetal adverse effects of our health intervention given the importance of such effects in the implementation process. We describe our KT strategy and targeted sites, outcome measurement and data analysis using data from the Canadian Perinatal Network (CPN) and Canadian Neonatal Network (CNN).

## Methods

### KT strategy (2011–2015) and targeted centres

We undertook an interrupted time-series study design using segmented regression analysis to evaluate the effectiveness of a selected bundle of KT strategies to optimise use of MgSO4 for fetal NP. We have employed the use of the StaRI (Standards for Reporting Implementation Studies) as our reporting standard [27].

**Fig. 1:**
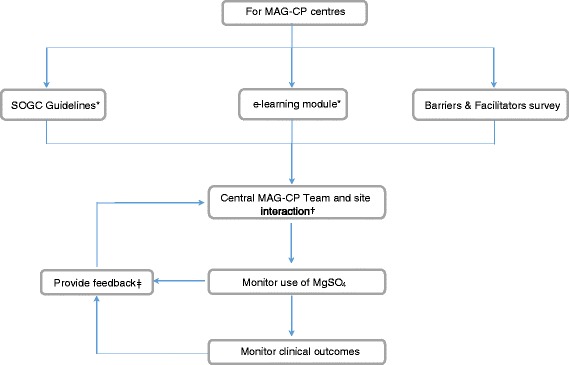
Schematic of the MAG-CP knowledge translation audit cycle. *All members of the Society of Obstetricians and Gynaecologists of Canada (SOGC) were sent the guideline and link to the e-learning module, both of which were open-access to anyone else who was made aware of them. †Central MAG-CP team interactions with each site included site visits to study centres where members of the central team presented didactic grand rounds and facilitated small site-specific interactive group discussion; a monthly newsletter; monthly teleconferences; supportive emails and one-on-one support for questions and advice; provision of KT tools (such as pre-printed physician orders, presentation materials, information sheets for staff and women; and reminders for women who were being expectantly managed in hospital and at risk of preterm birth at < 32 weeks [www.cpn-rpc.org]). ǂFeedback included semi-annual site-specific reports on MgSO4 for fetal NP use that compared each site with activity overall (while maintaining the anonymity of other sites), thus creating an audit cycle to inform and fuel ongoing KT

The strategy consisted of four parts: (1) initiating and leading a Society of Obstetricians and Gynaecologists of Canada (SOGC) clinical practice guideline on the topic that was published in May 2011 [26] and then from 2011 to 2015 (as previously detailed [16]), (2) an e-learning module; (3) a ‘Barriers and Facilitators Survey’ and (4) an audit and feedback cycle, including site visits, monitoring and other interactive activities between the central MAG-CP team and individual sites. We have previously published a qualitative analysis of our strategies [16] (Fig. 1).

The first two components of the KT strategy were available to all practitioners in Canada. The SOGC guidelines were open-access and free to anyone who received the Society’s journal or had internet access. The e-learning module was created by the central MAG-CP team, published by AdvancingIn®, an online platform providing electronic continuing medical education, which was free to all Canadian health care professionals who were SOGC members. The module was developed by the senior authors of the guideline on behalf of the SOGC, who separately contracted AdvancingIn® for their platform. This module included pre-test questions, a concise summary of the evidence and the 2011 SOGC guideline, a summary of controversies and uncertainties, case analyses, practice tools, post-test questions, and a discussion forum regarding MgSO4 for NP. The module was incentivised by providing continuing medical education (CME) credits to eligible health care professionals and a certificate of completion.

The third and fourth components of the KT were delivered to practitioners at participating study sites in the Canadian Perinatal Network (CPN) that agreed to participate in the KT activities and collect relevant outcome data (see below). These centres were tertiary perinatal centres that were likely to see women eligible for the intervention. The ‘Barriers and Facilitators (B&F) survey’, informed by the Theoretical Domains Framework [28], was distributed at each MAG-CP study participating site, by each site’s local team to be completed by at least five obstetricians and five nurses (to explore local barriers to and facilitators of MAG-CP implementation). The surveys were anonymised and consisted of mixed free text and tick choices, collected locally, and sent to the central team for compilation and interpretation to provide feedback of results to each site for their review. This approach was chosen to determine organisation readiness and address challenges as well as identify knowledge gaps and tailor interventions. Further details and a copy of the survey have been published in our detailed qualitative analysis [16].

The audit and feedback cycle to address local barriers consisted of visits to study centres that were organised by the central team. They presented didactic grand rounds and facilitated small site-specific interactive group discussion. Other activities for feedback and exploration of barriers included a monthly newsletter, monthly teleconferences, supportive emails and one-on-one support for questions and advice and provision of KT tools (presentation materials, information sheets for staff and women and reminders for women who were being expectantly managed in hospital and at risk of preterm birth at < 32 weeks [29]). As part of the feedback, the central MAG-CP team prepared 6-month site-specific reports on MgSO4 for fetal NP use that compared each site with activity overall (while maintaining the anonymity of other sites), thus creating the audit cycle to inform and fuel ongoing KT.

Interventions by the central MAG-CP team (such as e-learning module, site visits, completeness of B&F surveys and number of teleconferences participated) were directly measured as a form of fidelity of the KT strategies, while sites were asked to record other local KT strategies (that reflected participant responsiveness and other potential moderators of the intervention-adherence relationship [30]) using a web-based form (such as use of decision-aid tools, reminders, presentations or ‘teaching moments’). This was summarised as an ‘engagement’ measure of site participation in the intervention (Additional file 1: Table S7). ‘Highly engaged’ sites had values that were above the median or mean for each activity and overall; this assessment was conducted independently by each member of the MAG-CP working group (DAD, LAM and ARS), each of whom was masked to site identity. Discrepancies were resolved by consensus.

### Evaluation of KT strategies via the CPN

#### Enrollment into the CPN

To evaluate the effectiveness of the KT strategy, we tracked MgSO4 use and outcomes using data from the Canadian Perinatal Network (CPN) [31]. In brief, the CPN, another CIHR-funded project, collected demographic, management and outcome information (August 2005 and September 2015, inclusive) on women admitted at 22 weeks (and 0 days) to 28 weeks (and 6 days) with threatened very preterm birth to participating tertiary perinatal centres and who were followed to delivery. These women were admitted with one or more of spontaneous preterm labour with contractions, preterm pre-labour rupture of membranes (PPROM), short cervix without contractions, prolapsing membranes, gestational hypertension, intrauterine fetal growth restriction (IUGR) or antepartum haemorrhage (see Additional file 1: Table S2 for definitions). The project was approved centrally by the Research Ethics Board at the University of British Columbia (H05-70359 and H11-02214) and locally at each participating centre. As this was approved as a quality improvement project with no patient contact, patient consent was not deemed necessary.

#### Sample size calculation

Over the 4 years of the KT strategy, we anticipated recruitment of 3752 mothers based on previous CPN enrollment of women at < 29 weeks (from the CPN inception in 2005). We estimated that we would have > 95% power (two-sided alpha of 0.05, < 5% baseline use of MgSO4 for fetal NP) for each of two scenarios: (i) ‘planned’ rates of MgSO4 use for fetal NP of 20, 40, 60 and 80% by the end of years 1–4, respectively, and (ii) ‘pessimistic’ rates of 20, 30, 40 and 50% by the end of years 1–4, respectively, based on a prior survey with the centres. The power calculations were made without adjustment for random effects (i.e. clustering), because the calculations for these adjustments also require specification of the distribution of MgSO4 use across hospitals in the 4-year study period, and these were not known.

For adverse maternal outcomes, we estimated at least 80% power to detect potential increases in serious maternal adverse effects reported in RCTs: hypotension (RR 1.51 [1.09, 2.09] from baseline of 6.5%), infusion stopped due to adverse effects (RR 2.81 [2.01, 3.93] from 2.6%), respiratory depression (RR 1.31 [0.83, 2.07] from 1.9%) and pulmonary oedema (RR 2.79 [0.74, 10.47] from 0.3%) [26].

No increase or decrease in stillbirth or neonatal death was anticipated, but we were powered to detect only substantial increases in these outcomes (i.e. an increase of 27–28% in total paediatric mortality under the planned and ‘pessimistic’ pre-specified rates of MgSO4 use).

#### Sampling of eligible cases

Pregnancies were tracked in participating CPN sites in ‘pre-’ and ‘post-’ MAG-CP eras (dates inclusive): (i) ‘pre’-MAG-CP (‘controls’, August 2005 to May 2011) and (ii) ‘post’, termed MAG-CP (‘cases’, June 2011 to September 2015). The pre-MAG-CP era represented the period before the KT intervention, from (i) the beginning of CPN data collection through the publication of the last primary trial of MgSO4 for fetal NP (2005–2008) [5–8], (ii) the publication of three independent systematic reviews of those primary trials (2009) [9–11] and (iii) the period thereafter until publication of the Canadian SOGC Clinical Practice Guidelines on MgSO4 for fetal NP [26] in May 2011 (Jan 2010–May 2011). Data were included from all CPN sites. The MAG-CP era ran from June 2011 until Sept 2015 (sub-divided into nine 6-month time periods) and included data from CPN sites that chose to participate in the MAG-CP study.

CPN-eligible pregnancies were ≥ 24 weeks (and 0 days) at CPN enrollment, presented with imminent preterm birth (i.e. likely within 24 h) at < 32 weeks (and 0 days) (using criteria consistent with the relevant primary trials and as summarised in Canadian guidance [26]) and were followed to delivery. CPN pregnancies were excluded if women had received MgSO4 for an indication other than fetal NP (e.g. eclampsia prophylaxis or treatment).

#### Data collection and analysis of outcome

Maternal information collected from CPN included maternal characteristics, obstetric history, details of hospital admission, maternal and fetal surveillance, labour and delivery, maternal outcomes, other maternal interventions (including MgSO4 administration and indication), stillbirth and neonatal outcome (other than resuscitation which was not available in the CPN). As the CPN database was revised for MAG-CP to include detailed information on MgSO4 administration (including dose, duration and side effects), this information was only available for pregnancies during the MAG-CP era (2011–2015).

The primary outcome was the rate of optimal MgSO4 use (i.e. administration when and only when needed), over time. ‘Underuse’ was defined as failure to administer MgSO4 for fetal NP when indicated (i.e. for birth that occurred within 24 h of admission to hospital at < 32 weeks (and 0 days)) and suboptimal use as administration of MgSO4 for fetal NP when not indicated, either when birth did not occur within 24 h at < 32 weeks (and 0 days) or birth occurred at ≥ 32 weeks (and 0 days). Secondary outcomes monitored included adverse maternal and neonatal effects. Maternal adverse effects included hypotension (i.e. diastolic blood pressure fall of > 15 mmHg), the need to stop MgSO4 because of side effects (‘stopped infusion’), respiratory depression (i.e. < 12 breaths/min) and pulmonary oedema (as per the clinician’s assessment).

Descriptive statistics were used to summarise maternal characteristics, details of admission and outcomes, with chi-square or Mann-Whitney *U* test used where appropriate. As the primary outcome was the monthly rate of optimal MgSO4 use over time, we performed an interrupted time-series analysis, a powerful quasi-experimental study design, to evaluate the effect of the KT intervention in MAG-CP compared with pre-MAG-CP eras and distinguish it from any observed effects in the absence of intervention [32, 33]; segmented generalised estimating equations (GEE) logistic regression was used to account for centre variability. Also, MgSO4 use for fetal NP was compared between ‘highly engaged’ and ‘less engaged’ sites. To correct for pregnancies that may have been precipitous in nature, we adjusted for any administration of antenatal corticosteroids, reasoning that there would be enough time to administer MgSO4 for fetal NP if antenatal corticosteroids were administered. Sensitivity analyses were conducted using data only from centres that participated in both the pre- and MAG-CP eras to assess usage rates of MgSO4. All statistical analyses were performed using R statistical software [34]. A *p* value < 0.05 was considered statistically significant.

### Canadian Neonatal Network (CNN) data collection and analysis

To further explore trends in MgSO4 use for fetal NP in Canada and associated neonatal resuscitation rates, we obtained data from the CNN that collects information on babies admitted to neonatal intensive care units (NICUs) in 31 participating NICUs in Canada. We included babies born at 24 weeks (and 0 days) to 31 weeks (and 6 days) and collected information about use of MgSO4 for fetal NP as well as pregnancy characteristics. We examined the proportion of babies who received MgSO4 for fetal NP from January 2011 when ‘fetal NP’ was first listed as an indication for MgSO4 in the CNN database; as such, data were available for a portion of the pre-MAG-CP (January–May 2011, inclusive) and during MAG-CP (i.e. June 2011–September 2015, inclusive). Also, we examined rates of intensive neonatal resuscitation, defined by the CNN as the need for either (i) chest compressions or intubation and ventilation or (ii) epinephrine administration in the delivery room [35]. GEE logistic regression was used to examine whether MgSO4 use for fetal NP changed over time. Among babies exposed to MgSO4 for fetal NP (compared with those who did not receive MgSO4 or received it for an indication other than fetal NP), logistic regression was used to calculate the odds ratio (OR) for ‘intensive’ neonatal resuscitation. GEE was used to adjust for important covariates (i.e. multiple gestation, gender, gestational age at delivery, birth weight < 10th centile, outborn status, mode of delivery and antenatal corticosteroid use), and babies with congenital anomalies were excluded, as in prior CNN analyses [35]. A *p* value < 0.05 was considered statistically significant.

### Role of the funding source

The funder of the study had no role in study design, data collection, data analysis, data interpretation or writing of the report. The corresponding author as well as DAD and ARS had full access to all the data in the study and had final responsibility for the decision to submit for publication.

## Results

### Participating centres

Eighteen of Canada’s 23 tertiary perinatal centres contributed data to either the pre-MAG-CP or MAG-CP eras; nine centres contributed outcome data continuously from August 2005–September 2015, seven contributed only pre-MAG-CP and two contributed only during MAG-CP. Thus, eleven centres received the KT strategy from 2011 to 2015. Participating centres were from all geographic regions of Canada (i.e. 6 Western centres, 8 Ontario/Quebec centres and 4 Atlantic centres), with annual delivery volumes ranging from < 2000 to ≥ 5000. For details of site participation, see Additional file 1: Table S3.

Implementation fidelity was variable between sites, as reflected in our measure of ‘engagement’ with KT. Ten of 11 (90.9%) centres completed the e-learning module, and all centres completed the B&F surveys with a median of 15 respondents, as well as receiving audit and feedback. Eight centres received a site visit, covering 10/11 MAG-CP sites (Additional file 1: Table S7). One site visit could not be arranged at a mutually convenient time. Centres did not appear to be exposed or respond to the KT strategies equally.

### Outcomes—Canadian Perinatal Network data

There were 5683 women enrolled at 18 CPN sites during the pre-MAG-CP era, of whom 4745 (83.5%) were included for analyses. Similarly, there were 3784 women enrolled at 11 CPN sites (participating in MAG-CP) during the MAG-CP era, of whom 3143 (83.1%) were included. The proportion of eligible patients did not differ between the pre-MAG-CP and MAG-CP eras (*p* = 0.60).

### Characteristics of the sample

There were differences in the characteristics of women enrolled in pre-MAG-CP and MAG-CP eras (Table 1, which also presents maternal and perinatal outcomes for completeness). Many differences were small in magnitude (e.g. history of venous thromboembolism) and/or of questionable clinical significance (e.g. maternal age and gestational age at enrollment in CPN and at delivery). Overall, women were just over 30 years of age. Few women (< 5%) had pre-existing medical conditions. Among parous women, about one-third had experienced prior preterm delivery. Approximately half of women were nulliparous and almost 20% had multiple pregnancies. Most women were non-smokers in the current pregnancy, particularly during the MAG-CP era. Women were enrolled in CPN at about 26 weeks, usually for preterm labour and/or PPROM, and they delivered at about 30 weeks’ gestation. In terms of other maternal and perinatal outcomes, pregnancies in the MAG-CP era were more often complicated by abruption and serious maternal complications, although stillbirth and neonatal death were less frequent.

**Table 1:** Baseline characteristics and pregnancy outcomes of women with imminent preterm birth at participating Canadian Perinatal Network sites (2005–15) (*N* (%) women or median [IQR], unless otherwise stated)

	Total *N* = 7888	Pre-MAG-CP (2005–2011) *N* = 4745	MAG-CP (2011–2015) *N* = 3143	*p* values
Maternal demographics and past history				
Maternal age at EDD (year)	31 [27, 35]	31 [27,35]	31 [27,35]	0.034
Pre-existing medical conditions				
Pre-existing hypertension	305 (3.9%)	178 (3.8%)	127 (4.0%)	0.600
Diabetes mellitus	149 (1.9%)	79 (1.7%)	70 (2.2%)	0.090
Venous thromboembolism	28 (0.4%)	25 (0.5%)	3 (0.1%)	0.001
Prior obstetric history				
Previous preterm birth	1383 (17.5%)	845 (17.8%)	538 (17.1%)	0.400
Previous caesarean	930 (11.8%)	666 (14.0%)	264 (8.4%)	< 0.001
Current pregnancy				
Nulliparity	3909 (49.6%)	2303 (48.5%)	1606 (51.1%)	0.030
Multiple gestation	1507 (19.1%)	911 (19.2%)	596 (19.0%)	0.800
Smoking during pregnancy	1260 (16.0%)	833 (17.6%)	427 (13.6%)	< 0.001
Missing	52	33	19	
Gestational age at enrollment (week)	26.0 [24.4, 27.4]	26.1 [24.6, 27.6]	25.9 [24.4, 27.4]	< 0.001
Indication for threatened preterm birth				
Preterm labour only	2324 (29.5%)	1375 (29.0%)	949 (30.2%)	< 0.001
PPROM only	1567 (19.9%)	960 (20.2%)	607 (19.9%)	
PTL and PPROM	1106 (14.0%)	591 (12.5%)	515 (16.4%)	
Antepartum haemorrhage only	1195 (15.1%)	764 (16.1%)	431 (13.7%)	
Other†	1696 (21.5%)	1055 (22.2%)	641 (20.4%)	
Gestational age at delivery (week)	28.0 [26.0, 35.0]	28.0 [26.0, 35.0]	28.0 [26.0, 34.0]	0.036
≥ 37 weeks (and 0 days)	1404 (17.8%)	889 (18.7%)	515 (16.4%)	0.008
34 weeks (and 0 days)–36 weeks (and 6 days)	821 (10.4%)	507 (10.7%)	314 (10.0%)	
29 weeks (and 0 days)–33 weeks (and 6 days)	1522 (19.3%)	926 (19.5%)	596 (19.0%)	
< 29 weeks (and 0 days)	4141 (52.5%)	2423 (51.1%)	1718 (54.7%)	
Maternal outcomes				
Placental abruption after enrollment	635 (8.1%)	251 (5.3%)	384 (12.2%)	< 0.001
One/more serious maternal complications	2479 (31.4%)	1304 (27.5%)	1175 (37.4%)	< 0.001
Death	2 (0.03%)	2 (0.04%)	0	0.500
Admission to ICU or HDU	98 (1.2%)	19 (0.4%)	79 (2.5%)	< 0.001
Chorioamnionitis	1789 (22.7%)	999 (21.1%)	790 (25.1%)	< 0.001
Cardiovascular	2 (0.03%)	1 (0.02%)	1 (0.03%)	0.999
Respiratory	64 (0.8%)	38 (0.8%)	26 (0.8%)	0.999
CNS	7 (0.09%)	5 (0.1%)	2 (0.06%)	0.700
Renal	6 (0.08%)	4 (0.08%)	2 (0.06%)	0.999
Hematological	72 (0.9%)	37 (0.8%)	17 (0.5%)	0.300
Hepatic	11 (0.1%)	11 (0.2%)	0	0.004
Infection	74 (0.9%)	50 (1.1%)	24 (0.8%)	0.200
Perinatal outcomes	*N* = 9541	*N* = 5751	*N* = 3790	
Stillbirth	291 (3.0%)	211 (3.7%)	80 (2.1%)	< 0.001
Neonatal death in the delivery room	164 (1.7%)	119 (2.1%)	45 (1.2%)	0.002
Liveborn and admitted to NICU	7638 (80.1%)	4714 (82.0%)	2924 (77.2%)	<0.001

*PPROM* preterm premature rupture of membranes, *CNS* central nervous system, *HDU* high-dependency unit, *ICU* intensive care unit, *IQR* interquartile range, *NICU* neonatal intensive care unit

†Other indications for threatened preterm birth in the absence of preterm labour, PPROM or antepartum haemorrhage included (not mutually exclusive) gestational hypertension (*N* = 227), intrauterine growth restriction (*N* = 238), short cervix (*N* = 476), prolapsed membranes (*N* = 249) or other non-CPN condition within the Maternal-Infant Care Network (*N* = 48) in the pre-MAG-CP era and gestational hypertension (*N* = 137), intrauterine growth restriction (*N* = 151), short cervix (*N* = 325) or prolapsed membranes (*N* = 157) in the MAG-CP era

### Analysis of MgSO4 usage

MgSO4 for fetal NP was administered (either ‘optimally’ to those who needed it or ‘suboptimally’ to those who did not need it, as previously defined, see the ‘Methods’ section) to 94 (2.0%) of women in the pre-MAG-CP era and 1454 (46.3%) during MAG-CP. Details of MgSO4 administration were collected only during MAG-CP.

During the MAG-CP era, women received MgSO4 for fetal NP at about 27 weeks, approximately 1 week after admission with threatened very preterm birth (Table 2). Almost 40% of women were in active labour with ≥ 4 cm of cervical dilatation at the time of receiving MgSO4. More than 90% of women received one course of MgSO4 for fetal NP, with 82.4% of women receiving both loading dose and maintenance doses. The usual loading dose was 4 g iv over a median of 25 min. The median maintenance dose was 1 g/h iv for 7.4 h. MgSO4 was associated with few adverse effects. There were eight episodes of hypotension among seven women (0.5%) following loading (*N* = 5 episodes) or maintenance dosing (*N* = 3); none of the seven women had their infusions decreased or stopped or calcium gluconate administered. No woman experienced respiratory depression. Eleven women (0.7%) experienced pulmonary oedema. Other side effects (unspecified) occurred in one additional woman (0.1%) who was given calcium gluconate. Most women had their MgSO4 infusions stopped because they either delivered or were no longer considered at risk of imminent preterm birth.

**Table 2:** Details of 1512 women who received MgSO4 for fetal NP administration and adverse effects during MAG-CP (*N* (%) women unless otherwise specified)

	*N* (%) or median [IQR]
Gestational age at time of MgSO4 therapy (week)	27.1 [25.6, 28.4]
Cervical dilatation at time of therapy (cm)	3 [1.5,4.5]
Cervical dilatation ≥ 4 cm	570 (37.7%)
Cervical dilatation ≥ 4 cm among women with PTL	384/728 (52.7%)
Missing/unknown	227 (15.0%)
*N* treatment courses/woman	1 [1,1]
Received more than one course	110 (7.3%)
Received only loading dose	222 (14.7%)
Received only maintenance dose	44 (2.9%)
Received both loading and maintenance doses	1246 (82.4%)
Loading dose details	
Route of administration	
IV only	1464 (96.8%)
IM only	2 (0.1%)
Initial dose (g)	4 [4,4]
Duration of therapy (min)	25 [20, 30]
Missing	95 (6.5%)
Adverse maternal effects (one/more)	6 (0.4%)
Maternal hypotension	5 (0.3%)
Respiratory depression	0
Pulmonary oedema	1 (0.1%)
Loading dose stopped early	45 (3.1%)
Stopped because woman delivered	38 (2.6%)
Stopped because patient refused treatment or further treatment	1 (0.1%)
Stopped because of maternal side effects	0
Stopped because woman was no longer in imminent preterm birth	1 (0.1%)
Other*	4 (0.3%)
Calcium gluconate administered	0
Maintenance dose details	
Route of administration	
IV only	1290 (85.3%)
IM only	0
Initial dose (g/h)	1 [1,1]
Duration of therapy (h)	7.4 [3.1, 17.5]
Adverse maternal effects (one/more)	13 (0.9%)
Maternal hypotension	3 (0.2%)
Respiratory depression	0
Pulmonary oedema	10 (0.7%)
Reasons for stopping maintenance dose	
Stopped because woman delivered	738 (57.2%)
Stopped because 24 h of therapy had been administered	81 (6.3%)
Stopped because woman was no longer in imminent preterm birth	163 (12.6%)
Stopped because of maternal side effects	0
Other†	29 (2.2%)
No reason indicated or missing	279 (21.6%)
Calcium gluconate administered	1 (0.1%)

*CPN* Canadian Perinatal Network

*Other reasons for stopping the loading dose of MgSO4 for fetal NP early were emergency caesarean (*N* = 1), patient in extreme pain from IV (*N* = 1), patient felt burning/flushing (*N* = 1) and unknown (*N* = 1)

†Other reasons for stopping the maintenance dose of MgSO4 for fetal NP were as per protocol or other orders (e.g. 12 h of therapy administered) (*N* = 9), dosage change (*N* = 7), patient transferred (*N* = 4), MgSO4 continued postpartum for pre-eclampsia prevention (*N* = 3), fetal demise (*N* = 2), emergency caesarean (*N*= 2) or patient experienced side effects (*N* = 2)

### Segmented regression analysis

**Fig. 2:**
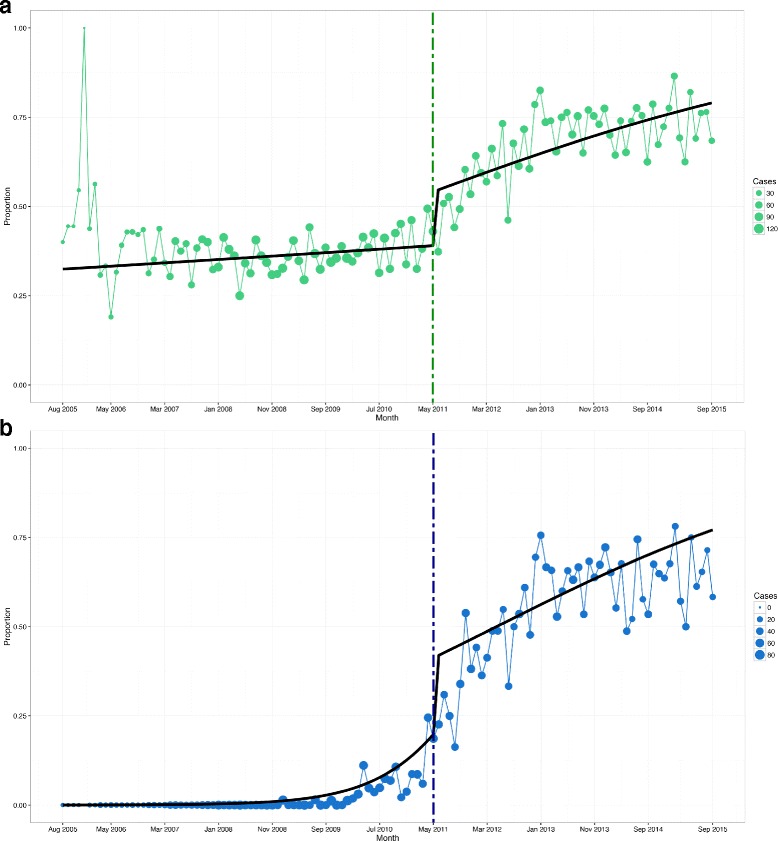
Segmented regression analysis of pre-MAG-CP (2005–11) and MAG-CP (2011–2015) eras. **a** Optimal use. **b** Appropriate use. The dashed line indicates implementation of the KT intervention

Table 3 presents the odds ratios of MgSO4 for fetal NP in the pre-MAG-CP and MAG-CP eras, according to optimal, under- and suboptimal use among eligible women. The absolute rates are presented in Additional file 1: Table S4, including the number of women in the optimal use category who needed MgSO4 for fetal NP and got it. In the pre-MAG-CP era, the odds of optimal use were increasing by 0.4% (OR 1.004 [0.997–1.01]) per month. Upon the start of the KT intervention, there was an immediate 84% increase in the odds of optimal use (OR 1.84 [1.51–2.24]), after which, there was a significant continuous increase of 2% (OR 1.02 [1.00, 1.04]) per month, compared to the pre-MAG-CP era (*p* < 0.001) (Fig. 2a). Thus, there was a 220% increase in the odds of optimal use during the MAG-CP era (OR 3.20); by comparison, the anticipated increase in optimal use, assuming that the KT intervention had not occurred, would be only 23% (OR 1.23). This increase in optimal use is mirrored by a fall in underuse.

**Table 3:** Overall odds ratios for use of MgSO4 for fetal NP as derived from segmented regression analysis

	Optimal use*	*p* value	Underuse*	*p* value	Suboptimal use*	*p* value
Odds ratio for use in pre-MAG-CP, per month†	1.004 [0.997, 1.01]	0.226	0.995 [0.99, 1.00]	0.104	1.18 [1.08, 1.28]	< 0.001
Immediate change in odds just after intervention†	1.84 [1.51, 2.24]	< 0.001	0.47 [0.34, 0.65]	< 0.001	2.18 [1.04, 4.58]	0.038
Change in odds ratio after intervention compared to pre-MAG-CP, per month†	1.02 [1.00, 1.04]	0.044	0.97 [0.95, 0.99]	0.002	0.86 [0.79, 0.94]	< 0.001
Odds ratio for use in MAG-CP era, per month†	1.02 [1.01, 1.03]	< 0.001	0.97 [0.95, 0.98]	< 0.001	1.01 [1.001, 1.02]	0.027

*Optimal use refers to both women who received MgSO4 for fetal NP when indicated, as well as women who did not receive MgSO4 for fetal NP when it was not indicated. Underuse refers to eligible women who should have received MgSO4 for fetal NP but did not. Suboptimal use refers to women who received MgSO4 too early (not within 24 h before birth) or at ≥ 32 weeks. †Segmented regression analysis was adjusted for antenatal administration of corticosteroids

**Fig. 3:**
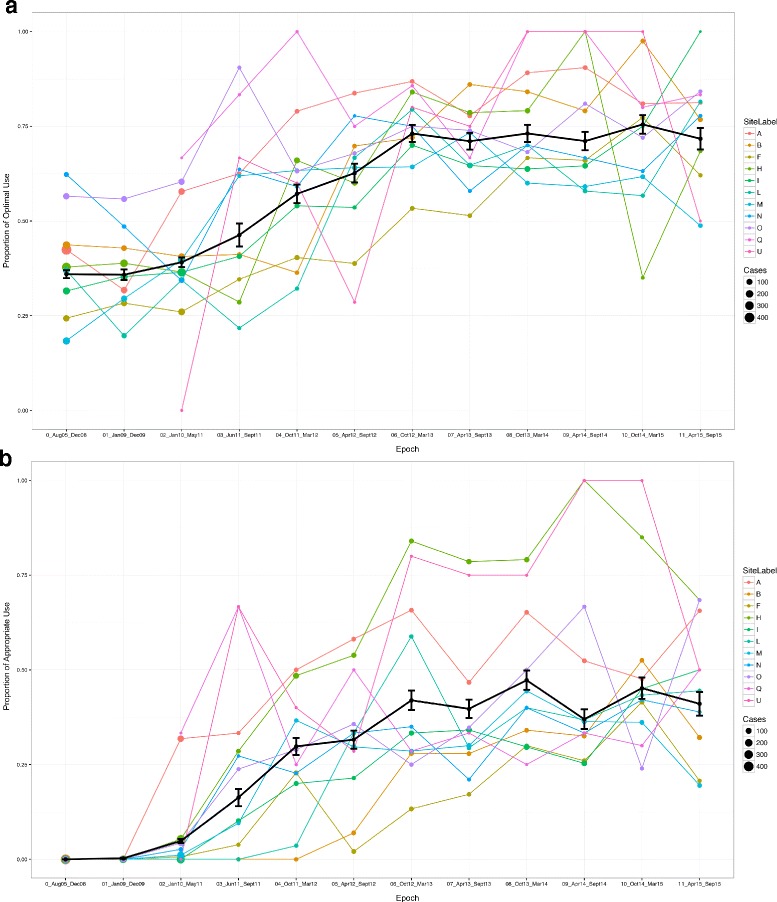
Absolute utilisation rates over pre-MAG-CP (2005–11) and MAG-CP (2011–2015) eras. **a** Optimal use. **b** Appropriate use. The solid black line indicates the median overall rate and each coloured line represents one of the 11 participating centres

The initial optimal use rate (36.0%) was related to non-administration to women for whom MgSO4 for fetal NP was not indicated (i.e. 751/2088, 36.0%), rather than administration to women for whom MgSO4 for fetal NP *was* indicated (i.e. 0/2088, 0%) (Fig. 3a, b). The odds of administration of MgSO4 for fetal NP to eligible women, termed ‘appropriate’ use, significantly increased upon the start of the KT intervention (*p* < 0.001) (Fig. 2b) and within the MAG-CP era specifically (*p* < 0.001).

Suboptimal use of MgSO4 for fetal NP also increased immediately after the KT intervention (OR 2.18, *p* = 0.038; Table 3) and continued to increase per month in the MAG-CP era but at a slower rate than in the pre-MAG-CP era. However, absolute rates remained quite low (< 13%) throughout the study (Additional file 1: Table S4). Although the SOGC guideline recommended only in the text (rather than in the recommendations) to consider MgSO4 for fetal NP for women between 32^+0^ and 33^+6^ weeks’ gestation with imminent preterm birth, few such women (79/3143, 2.5%) received such treatment during the MAG-CP era.

Sensitivity analyses based on the nine centres (7066 women) that contributed data to both pre-MAG-CP and MAG-CP eras were similar to the overall results (Additional file 1: Table S5 and S6).

**Fig. 4:**
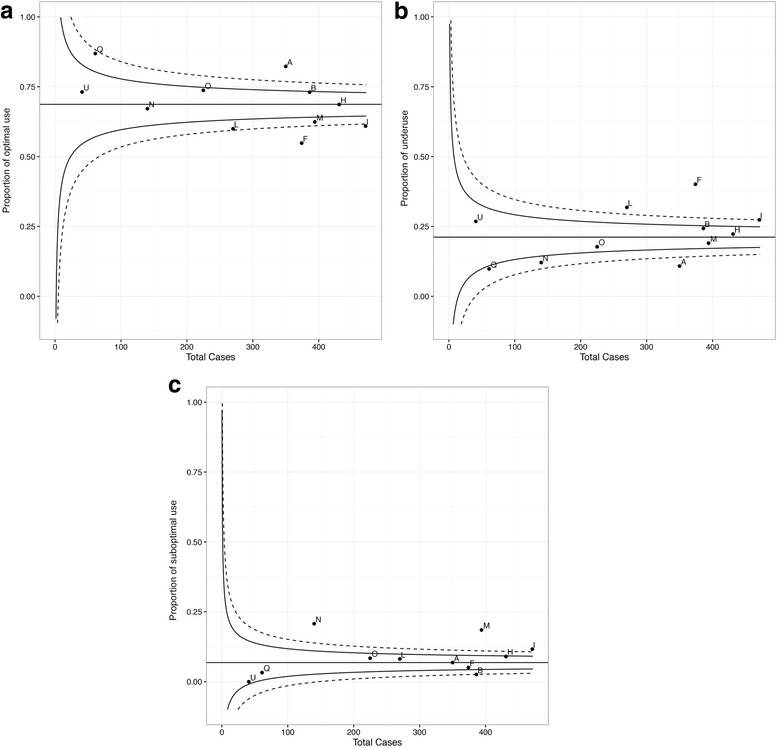
Between-centre variability of MgSO4 usage among centres. **a** Variability in optimal use. **b** Variability in underuse. **c** Variability in suboptimal use. The solid line indicates 95% confidence interval while the dotted line indicates the 99% confidence interval

Despite the strategies, there was substantial between-centre variability in optimal use and underuse rates of MgSO4 for fetal NP (Fig. 4a, b); two sites had optimal use rates ≥ 95th centile and four sites rates ≤ 5th centile. In general, sites with high optimal rates had lower absolute underuse rates and vice versa. There was far less variability seen in suboptimal use (Fig 4c); three of 11 sites had suboptimal use rates that were ≥ 95th centile, most often related to use among women who did not deliver imminently (i.e. within 24 h, 213/3143 [6.8%]) (Additional file 1: Table S4).

### Site engagement

Seven of 11 MAG-CP sites were ranked as ‘highly engaged’ and four as ‘less engaged’, based on criteria listed in Additional file 1: Table S7. However, neither this overall measure of engagement, nor the individual components of KT on which the assessment was based, including those directly measurable by the central team and reflecting implementation fidelity, were associated with optimal use ≥ 95th centile, although there appeared to be a strong trend towards such high optimal use and early ‘buy-in’ by participation in MAG-CP data collection (Additional file 1: Table S8).

#### Canadian Neonatal Network (CNN) data

Among 14,108 infants born at 24–31 completed weeks and admitted to NICU in 31 CNN sites (January 2011–September 2015), there was a significant increase in use of MgSO4 for fetal NP over time, from 19.7% pre-MAG-CP (Jan 01, 2011 to May 31, 2011) to 62.4% in the last MAG-CP time period (Apr 1, 2015 to Sept 30, 2015) (*p* < 0.001; Additional file 1: Table S9). There was, however, substantial between-centre variability in the use of MgSO4 for fetal NP, and optimal use ≥ 99th percentile was more frequent at MAG-CP sites (8/11) than at other CNN sites (4/19, *p* = 0.015; Additional file 1: Figure S3). No sites demonstrated optimal use that was between the 5th and 95th percentiles.

Antenatal and birth characteristics of infants differed between infants who were either unexposed, exposed for fetal NP or exposed for other indications (Table 4). Following adjustment for multiple gestation, gender, gestational age at delivery, birth weight < 10th centile, outborn status, mode of delivery and antenatal corticosteroid use, MgSO4 for fetal NP was associated with a lower risk of intensive neonatal resuscitation compared with either (i) non-receipt of MgSO4 (adjusted OR 0.63 [0.54, 0.73], *p* < 0.001) or (ii) receipt of MgSO4 for an indication other than fetal NP (adjusted OR 0.81 [0.66, 0.99], *p* = 0.04).

**Table 4:** Selected infant characteristics and outcomes according to exposure to MgSO4 for fetal NP and its indication (*N* (%) or median [IQR], where appropriate, unless otherwise indicated)

	MgSO4 for neuroprotection *N* = 5314	No MgSO4 *N* = 7238	MgSO4 for another indication *N* = 1556	*p* value
Antenatal and birth characteristics				
GA, weeks and days	29 [26, 30]	29 [27, 30]	29 [27, 30]	< 0.001
24 weeks (and 0 days)–28 weeks (and 6 days)	2634 (49.6%)	3198 (44.2%)	682 (43.8%)	< 0.001
29 weeks (and 0 days)–31 weeks (and 6 days)	2680 (50.4%)	4040 (55.8%)	874 (56.2%)	
Male	2908/5304 (54.8%)	3974/7233 (54.9%)	766 (49.9%)	< 0.001
Missing	10	5	3	
Singleton	3644 (68.6%)	5095 (70.4%)	1189 (76.5%)	< 0.001
Missing	0	1	1	
Small gestational age	545 (10.3%)	541 (7.5%)	277 (17.8%)	< 0.001
Missing	8	5	3	
Maternal chorioamnionitis	995 (23.5%)	1046 (21.0%)	168 (15.0%)	< 0.001
Missing	1085	2264	439	
Antenatal corticosteroids	5127 (96.9%)	5827 (81.7%)	1477 (95.8%)	< 0.001
Missing	23	108	14	
Outborn	299 (5.6%)	1484 (20.5%)	128 (8.2%)	< 0.001
Missing	0	6	2	
Delivered by caesarean	3082 (58.1%)	4260 (59.0%)	1119 (72.2%)	< 0.001
Missing	7	13	5	
Intensive resuscitation	1630 (30.9%)	2828 (39.6%)	466 (30.3%)	< 0.001
Missing	33	91	19	
Adjusted OR (vs no use)*	0.63 [0.54, 0.73]	Reference	–	< 0.001
Adjusted OR (vs other use)*	0.81 [0.66, 0.99]	–	Reference	0.04

*Adjusted for gestational age, gender, small for gestational age, singleton, outborn status, delivery by caesarean and administration of corticosteroids. Chorioamnionitis was not included due to a large proportion of missing variables

## Discussion

### Summary of results

In an interrupted time-series analysis of a large cohort of women who were enrolled in the CPN following admission with threatened preterm birth and who were eligible for MgSO4 for fetal NP, we found that our multifaceted KT strategy resulted in a significant increase in optimal use of MgSO4 for fetal NP. This was primarily due to an increase in administration to women who were eligible to receive it, with a minimal increase in administration to women who went on to deliver more than 24 h later or at ≥ 32 weeks (and 0 days). Although women in the pre-MAG-CP and MAG-CP eras differed according to a number of maternal and pregnancy characteristics, none would influence administration of MgSO4 for fetal NP and could be expected to account for the trends observed in MgSO4 use, particularly as these differences over time were observed only during the MAG-CP era.

In addition, using data from the CNN, tertiary perinatal centres that participated in MAG-CP (compared with those that did not) had higher optimal use rates of MgSO4 for fetal NP. Finally, MgSO4 for fetal NP was safe, with few maternal side effects (documented in MAG-CP centre) despite women having pregnancies more frequently complicated by placental abruption and other maternal complications during the MAG-CP era and no increase in neonatal resuscitation (demonstrated in CNN sites).

### How the findings fit with the published literature

#### KT approach

Multifaceted KT approaches can be more effective than dissemination alone in encouraging the adoption and implementation of new research results [36], changing clinical outcomes [37–40] and achieving improvements in policy and practice [41]. Specifically, audit and feedback are effective enablers of evidence-based guideline implementation [42]. While older reviews found multiple KT interventions to be more effective than single-strategy approaches [43–45], this is not necessarily the case in more recent literature in which single interventions can have an impact similar to multifaceted approaches [46]. However, this may vary according to the circumstances, such as the complexity of the health intervention or the organisational culture in which the intervention is implemented [19, 36]. This was what we found in a published analysis of the relative merits of our online e-learning module, interactive site visits (with educational rounds and focus group discussions) and circulation of an anonymous Barriers and Facilitators survey to systematically identify barriers to and facilitators of practice change [16]. In brief, no individual KT method was superior to the others with regards to (i) breadth of respondents reached; (ii) rates and richness of identified barriers, facilitators, and knowledge needed; and (iii) cost, in combination. The e-learning module reached the most diverse audience of health care providers, the site visits provided opportunity for iterative dialogue and the survey was the least expensive. Although the site visits provided the most detailed information around individual and organisational barriers, the ‘Barriers and Facilitators’ survey provided more detail about social-level barriers.

We recognise that costs and resources have implications. The bulk of our resources were used in the collection of outcome data. If health care systems routinely collected information related to monitoring the outcome, the cost of the project would consist of creation of resources and support of the local KT teams to move it forward.

#### Health intervention

Many international societies and bodies have now issued clinical practice guidelines that recommend MgSO4 for fetal NP in the setting of imminent preterm birth at < 32–34 weeks [26, 47–49]. The implementation of MgSO4 for fetal NP has been evaluated in single centres in the USA (that led the BEAM trial) [50], France [51], New Zealand [52] and Australia, where a similar multicentre KT implementation project is ongoing [53]. Although final analyses and follow-up results of the latter study are still pending, preliminary results in one institution (where the Australian primary trial was undertaken) showed a fall in underuse rates from 69.7 to 26.9% over 2 years [54]. Although we are not aware of other such KT initiatives in relation to MgSO4, the need for them has been recognised [55]. Studies of international practice confirm practice heterogeneity [17, 56, 57]).

Historically, MgSO4 has been regarded as increasing the risk of neonatal respiratory depression, hypotonia and the need for resuscitation [58]. However, our finding that use of MgSO4 for fetal NP does not increase (but rather is associated with a decrease in) the need for intensive neonatal resuscitation at delivery is consistent with more recent literature that has demonstrated no increase in resuscitation [13, 59–61] and, in some cases, a *decreased* need [35]. To date, no adverse effects of MgSO4 for fetal NP have been demonstrated on fetal heart rate [12], and reassuring results have been published from neurodevelopmental follow-up (including intelligence quotient [IQ] measurement) of children from the original NP trials [62, 63].

### Strengths and limitations of the data

To our knowledge, this is the first multicentre KT initiative of MgSO4 for fetal neuroprotection and of implementation of national SOGC guidelines using a multifaceted strategy. The CPN and MAG-CP datasets represent a large population of women who presented with threatened preterm birth, and the sites that have contributed data represent most (78%) Canadian tertiary perinatal centres, of various sizes and from different geographic regions. KT activities were well-documented prospectively and over a sufficient period of time (i.e. 11 years) so that trends in MgSO4 use for fetal NP could be analysed according to critical KT events. We used an interrupted time-series analysis, which is a powerful quasi-experimental study design, to evaluate the effect of the KT intervention and distinguish it from any observed effects in the absence of intervention [32, 33]. Our prospective data collection in the CPN was detailed, included timing and dosage of MgSO4, pregnancy characteristics and maternal complications and fetal and neonatal outcomes. Also, we included data from the CNN, which allowed us to expand our analyses to non-MAG-CP sites and examine the impact (adjusted for confounders) of MgSO4 for fetal NP on delivery room intensive neonatal resuscitation, an outcome not available in the CPN. As such, we believe our KT findings are generalisable to other clinicians who administer MgSO4 and manage threatened very preterm birth, decision makers and researchers wishing to implement a national maternity care clinical practice guideline or change practice.

Among our limitations is the fact that site investigators had to report some (but not all) local KT activities, raising the possibility that some activities affecting MgSO4 use may have been either over-reported or missed. Second, although our sample size of women was large overall, when examining effects within individual sites, or the association between individual components of the KT bundle compared with the overall bundle, we lacked power [64]; we were unable to confirm which strategies were responsible for the change in practice reported. Further, some aspects of the KT intervention were applied at the same time across all centres (i.e. the SOGC guidelines, the e-learning module and invitations to central MAG-CP activities, such as newsletters and monthly teleconferences); however, the application of other aspects of KT were applied at different (non-random) times across sites (such as site visits and local rounds). It is also possible that these KT strategies worked synergistically rather than the sum of effects by its individual components [65]. Nevertheless, our segmented regression analysis shows an increase in the optimal use during the KT intervention period. Moreover, the CNN data (from 31 sites) indicated that sites that participated in MAG-CP (vs those that did not) had higher optimal use rates. Third, relating the maternal adverse effects and neonatal resuscitation outcomes of MgSO4 in the same population of subjects would have been optimal; however, we were unable to directly link MAG-CP data with CNN.

## Conclusions

Optimal use of MgSO4 for fetal NP in Canada increased significantly over 4 years with a multifaceted KT strategy that included education, engagement of health care professionals and identification of barriers and facilitators by the local team. We have demonstrated that it is possible to move from evidence-based national policy to implementation. Our central support of the local KT teams is a model worthy of consideration when planning implementation of other clinical practice guidelines, whether local, regional or national. Specific to MgSO4 for fetal NP, future work should explore between-centre variability in practice, the resolution of which may aid in achieving the target of 80% optimal use of MgSO4 for fetal NP. We await the results of pediatric motor and neurodevelopmental outcomes associated with antenatal MgSO4 for fetal NP that are being tracked by the Canadian Neonatal Follow-Up Network at CPN and CNN sites [66].

In general, future work should explore which components of a multifaceted strategy are particularly useful for implementing certain types of health interventions, such as drug interventions or surgical manoeuvres.

## Additional file

Additional file 1: Figure S1. Between-centre variability of optimal, under and suboptimal uses of MgSO4 among the 11 participating centres. **Figure S2.** Trend of antenatal corticosteroid administration over time among women with underuse of MgSO4 as a proxy for non-precipitous deliveries. **Figure S3.** Variability of MgSO4 use for fetal neuroprotection among MAG-CP centres (represented by the blue triangles) and non-MAG-CP centres (represented by the green circles). **Table S1.** MAG-CP (MAGnesium sulphate for fetal neuroprotection to prevent Cerebral Palsy), CPN (Canadian Perinatal Network) and CNN (Canadian Neonatal Network) collaborative groups. **Table S2.** Definitions of conditions and variables as used in the Canadian Perinatal Network (CPN). **Table S3.** Geographic regions of participating centres in the Canadian Perinatal Network (CPN). **Table S4.** Absolute utilisation rates of MgSO4 for fetal NP by study time period (from August 01/05 to September 30/15). **Table S5.** Segmented regression analysis of the nine centres that contributed data to both pre-MAG-CP and MAG-CP eras. **Table S6.** Sensitivity analyses of overall utilisation rates of MgSO4 using data from the nine centres that contributed data to both pre-MAG-CP and MAG-CP eras. **Table S7.** Determinants of engagement of participating sites in MAG-CP. **Table S8.** Components of engagement and relation to optimal use. Table S9. Antenatal MgSO4 use at GA 24–31^+6^ weeks by indication, from Jan 1/11 to Sep 30/15. (DOCX 281 kb)

## Abbreviations

B&F: Barriers and Facilitators
CIHR: Canadian Institutes of Health Research
CNN: Canadian Neonatal Network
CP: Cerebral palsy
CPN: Canadian Perinatal Network
GEE: Generalised estimating equations
IUGR: Intrauterine growth restriction
KT: Knowledge translation
MAG-CP: MAGnesium sulphate for fetal neuroprotection to prevent Cerebral Palsy
MgSO4: Magnesium sulphate
NICU: Neonatal intensive care unit
NP: Neuroprotection
PPROM: Preterm pre-labour rupture of membranes
SOGC: Society of Obstetricians and Gynaecologists of Canada
