# Supplementary material for: MAGnesium sulphate for fetal neuroprotection to prevent Cerebral Palsy (MAG-CP)—implementation of a national guideline in Canada
Source: Implement Sci. 2018 Jan 11;13:8. doi: 10.1186/s13012-017-0702-9 (PMC5765609; doi:10.1186/s13012-017-0702-9)
Supplement: Additional file 1: Figure S1. — Between-centre variability of optimal, under and suboptimal uses of MgSO4 among the 11 participating centres. Figure S2. Trend of antenatal corticosteroid administration over time among women with underuse of MgSO4 as a proxy for non-precipitous deliveries. Figure S3. Variability of MgSO4 use for fetal neuroprotection among MAG-CP centres (represented by the blue triangles) and non-MAG-CP centres (represented by the green circles). Table S1. MAG-CP (MAGnesium sulphate for fetal neuroprotection to prevent Cerebral Palsy), CPN (Canadian Perinatal Network) and CNN (Canadian Neonatal Network) collaborative groups. Table S2. Definitions of conditions and variables as used in the Canadian Perinatal Network (CPN). Table S3. Geographic regions of participating centres in the Canadian Perinatal Network (CPN). Table S4. Absolute utilisation rates of MgSO4 for fetal NP by study time period (from August 01/05 to September 30/15). Table S5. Segmented regression analysis of the nine centres that contributed data to both pre-MAG-CP and MAG-CP eras. Table S6. Sensitivity analyses of overall utilisation rates of MgSO4 using data from the nine centres that contributed data to both pre-MAG-CP and MAG-CP eras. Table S7. Determinants of engagement of participating sites in MAG-CP. Table S8. Components of engagement and relation to optimal use. Table S9. Antenatal MgSO4 use at GA 24–31+6 weeks by indication, from Jan 1/11 to Sep 30/15. (DOCX 281 kb) [file 13012_2017_702_MOESM1_ESM.docx]

**SUPPLEMENTARY APPENDIX**

| **Table/figure** | **Title** | **Page** |
| --- | --- | --- |
| **Figure S1** | Variability in optimal, under, and suboptimal uses | 2 |
| **Figure S2** | Trend of antenatal corticosteroid administration among underuse cases | 3 |
| **Figure S3** | Variability in use of MgSO4 for fetal NP among CNN centres | 4 |
| **Table S1** | CPN, MAG-CP, and CNN Collaborative Groups | 5 |
| **Table S2** | Definitions of conditions and variables as used in the Canadian Perinatal Network | 7 |
| **Table S3** | Geographic regions of CPN centres | 8 |
| **Table S4** | MgSO4 utilization rates over time | 9 |
| **Table S5** | Sensitivity analyses of the change in odds of MgSO4 use over time | 10 |
| **Table S6** | Sensitivity analyses of MgSO4 utilization rates among MAG-CP sites only | 11 |
| **Table S7** | Determinants for highly vs less engaged centres | 12 |
| **Table S8** | Components of engagement and relation to optimal use. | 13 |
| **Table S9** | Adjusted analyses for intensive resuscitation | 14 |

**Figure S1**. **Between-centre variability of optimal, under, and suboptimal uses of MgSO4 among the 11 participating centres**

**Figure S2. Trend of antenatal corticosteroid administration over time among women with underuse of MgSO4 as a proxy for non-precipitous deliveries.**


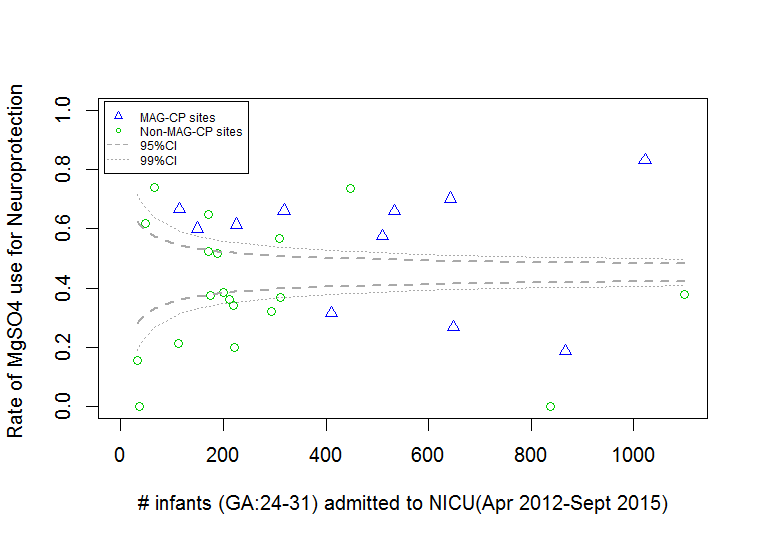


**Figure S3. Variability of MgSO4 use for fetal neuroprotection among MAG-CP centres (represented by the blue triangles) and non-MAG-CP centres (represented by the green circles).**

**Table S1: MAG-CP (MAGnesium sulphate for fetal neuroprotection to prevent Cerebral Palsy), CPN (Canadian Perinatal Network), and CNN (Canadian Neonatal Network) Collaborative Groups**

| **MAG-CP Collaborative Group** |
| --- |
| ***Steering Committee****:* Laura A. Magee (St. George’s University of London, London UK), Anne Synnes (University of British Columbia, Vancouver BC), Victoria M. Allen (Dalhousie University, Halifax NS), J. Mark Ansermino (University of British Columbia, Vancouver BC), François Audibert (Université de Montréal, Montreal QC), Rollin Brant (University of British Columbia, Vancouver BC), Emmanuel Bujold (Université Laval, Québec QC), Joan M.G. Crane (Memorial University of Newfoundland, St. John's NF), KS Joseph (University of British Columbia, Vancouver BC), Bruno Piedboeuf (Université Laval, Québec QC), Peter von Dadelszen (St. George’s University of London, London UK), Mark Walker (University of Ottawa, Ottawa ON), Wendy Whittle (University of Toronto, Toronto ON), Carmen Young (University of Alberta, Edmonton AB |
| ***Working Group:*** Laura A. Magee (Chair), Anne Synnes, Dane A. De Silva |
| ***Data Team****:* Larry Li (programmer), Tang Lee (analyst), Jeffrey N. Bone (analyst), Dane A. De Silva (analyst), KS Joseph (consultant), Rollin Brant (consultant) |
| *Site Investigators & Co-ordinators:* Laura A. Magee, Anne Synnes, Melanie Basso, & Diane Sawchuck (BC Women’s Hospital & Health Centre, Vancouver BC), Carmen Young & Cheryl Lux-Warholic (Royal Alexandra Hospital, Edmonton AB), Renato Natale, Laura McMurphy & Jennifer Ryder (London Health Sciences Centre, London ON), Wendy Whittle & Karen Muller (Mount Sinai Hospital, Toronto ON), Mark Walker & François Tshibemba (The Ottawa Hospital, Ottawa ON), François Audibert & Jocelyne Valée (Centre Hôspitalier Universitaire Sainte-Justine, Montréal QC), Emmanuel Bujold & Amélie Tétu (Centre Hôspitalier de L’Université Laval, Québec City QC), Victoria Allen & Venessa Ryan (IWK Health Centre, Halifax NS), Joan M.G. Crane, Donna Hutchens & Sandra Adams (Women's Health Program, Eastern Health, St. John’s NL), Kimberly Butt & Lynda Nicoll (Dr. Everett Chalmers Regional Hospital, Fredericton NB), and Lynn Murphy-Kaulbeck, Claire Williams & Norma Leger (The Moncton Hospital, Moncton NB). |
| CPN Collaborative Group |
| Laura A. Magee and Robert Liston (BC Women’s Hospital & Health Centre, Vancouver BC), Jerome Dansereau (Victoria General Hospital, Victoria BC), Carmen Young (Royal Alexandra Hospital, Edmonton AB), Stephen Wood (Foothills Medical Centre, Calgary AB), Femi Olatunbosun (Royal University Hospital, Saskatoon SK), George Carson, (Regina General Hospital, Regina SK), Graeme Smith (Kingston General Hospital, Kingston ON), Renato Natale (London Health Sciences Centre, London ON), Wendy Whittle (Mount Sinai Hospital, Toronto ON), Mark Walker (The Ottawa Hospital, Ottawa ON), Sarah McDonald (McMaster University Medical Centre), François Audibert (Centre Hôspitalier Universitaire Sainte-Justine, Montréal QC), Jean-Charles Pasquier (Centre Hôspitalier Universitaire de Sherbrooke, Sherbrooke QC), Emmanuel Bujold (Centre Hôspitalier de L’Université Laval, Québec City QC), Victoria M. Allen (IWK Health Centre, Halifax NS), Joan M.G. Crane (Women's Health Program, Eastern Health, St. John’s NL) |
| CNN Collaborative Group |
| Prakesh S Shah (CNN Director, Mount Sinai Hospital, Toronto, ON), Adele Harrison (Victoria General Hospital, Victoria, BC), Anne Synnes and Joseph Ting (British Columbia Women’s Hospital, Vancouver, BC), Zenon Cieslak (Royal Columbian Hospital, New Westminster, BC), Rebecca Sherlock (Surrey Memorial Hospital, Surrey, BC), Wendy Yee (Foothills Medical Centre, Calgary, AB), Carlos Fajardo (Alberta Children’s Hospital, Calgary, AB), Khalid Aziz and Jennifer Toye (Royal Alexandra Hospital, Edmonton, AB), Zarin Kalapesi (Regina General Hospital, Regina, SK), Koravangattu Sankaran and Sibasis Daspal (Royal University Hospital, Saskatoon, SK), Mary Seshia (Winnipeg Health Sciences Centre, Winnipeg, MB), Ruben Alvaro (St. Boniface General Hospital, Winnipeg, MB), Sandesh Shivananda (Hamilton Health Sciences Centre, Hamilton, ON), Orlando Da Silva (London Health Sciences Centre, London, ON), Chuks Nwaesei (Windsor Regional Hospital, Windsor, ON), Kyong-Soon Lee (Hospital for Sick Children, Toronto, ON), Michael Dunn (Sunnybrook Health Sciences Centre, Toronto, ON), Brigitte Lemyre (Children’s Hospital of Eastern Ontario and Ottawa General Hospital, Ottawa, ON), Kimberly Dow (Kingston General Hospital, Kingston, ON), Ermelinda Pelausa (Jewish General Hospital, Montréal, QC), Keith Barrington (Hôpital Sainte-Justine, Montréal, QC), Christine Drolet and Bruno Piedboeuf (Centre Hospitalier Universitaire de Québec, Québec City, QC), Daniel Faucher and Martine Claveau (McGill University Health Centre, Montréal, QC), Valerie Bertelle and Edith Masse (Centre Hospitalier Universitaire de Sherbrooke, Sherbrooke, QC), Rody Canning (The Moncton Hospital, Moncton, NB), Hala Makary (Dr. Everett Chalmers Hospital, Fredericton, NB), Cecil Ojah and Luis Monterrosa (Saint John Regional Hospital, Saint John, NB), Akhil Deshpandey (Janeway Children’s Health and Rehabilitation Centre, St. John’s, NL), Jehier Afifi (IWK Health Centre, Halifax, NS), Andrzej Kajetanowicz (Cape Breton Regional Hospital, Sydney, NS), Shoo K Lee (CNN Chairman, Mount Sinai Hospital, Toronto, ON). |

**Table S2. Definitions of conditions and variables as used in the Canadian Perinatal Network (CPN).**

| **Condition / Variable** | **Definition** |
| --- | --- |
| Preterm labour | Regular contractions every 5 minutes or more frequently, with documented cervical change at <37 weeks (and zero days) gestation. |
| Preterm pre-labour rupture of membranes (PPROM) | Pre-labour rupture of membranes confirmed by positive ferning and/or pooling of amniotic fluid prior to onset of regular contractions at <37 weeks (and zero days) gestation. |
| Short cervix without uterine contractions | Cervical shortening (≤10 mm by vaginal ultrasonography) without regular contractions (every 5 minutes or more frequently) |
| Dilated cervix or prolapsed membranes without uterine contractions | Prolapsed membranes at or beyond the external os as visualized on speculum exam, or any cervical dilatation of external os by endovaginal ultrasonography, without regular contractions. |
| Antepartum haemorrhage | >15 mL of vaginal bleeding prior to the onset of labour. |
| Gestational hypertension (GH) | Systolic BP ≥140 mmHg or diastolic BP ≥90 mmHg, twice, at least 4 hours apart, developing after 20 weeks. |
| Idiopathic intrauterine growth restriction (IUGR) | Ultrasonographically-determined abdominal circumference (AC) <10^th^ percentile by local definitions (e.g., provincial data, Jeanty chart), without a recognized cause other than GH |

**Table S3. Geographic regions of participating centres in the Canadian Perinatal Network (CPN)**

| **Centre Label** | **Geographic Region** |
| --- | --- |
| A | Western |
| B | Ontario/Quebec |
| C | Ontario/Quebec |
| D | Western |
| E | Western |
| F | Ontario/Quebec |
| G | Ontario/Quebec |
| H | Ontario/Quebec |
| I | Western |
| J | Western |
| K | Western |
| L | Ontario/Quebec |
| M | Ontario/Quebec |
| N | Eastern |
| O | Eastern |
| P | Ontario/Quebec |
| Q | Eastern |
| U | Eastern |

**Table S4. Absolute utilization rates of MgSO4 for fetal NP by study epoch (from August 01/05 to September 30/15)***

|  | **N** | **Optimal use** | | |  | **Underuse** |  | **Suboptimal use** | | |
| --- | --- | --- | --- | --- | --- | --- | --- | --- | --- | --- |
|  |  | **Use when indicated** | **No use when not indicated** | Total |  | **No use when indicated** |  | **Use that was too early before birth at <32wk** | **Use at ≥32wk** | Total |
| **Pre MAG-CP, by epoch (N)** | **4745** | **76** | **1676** |  |  | **3175** |  | **11** | **7** |  |
| Aug 01/05 to Dec 31/08 | 2088 | 0 | 751 (36.0%) | 36.0% |  | 1337 (64.0%) |  | 0 | 0 | 0% |
| Jan 01/09 to Dec 31/09 | 1145 | 3 (0.3%) | 407 (35.5%) | 35.8% |  | 735 (64.2%) |  | 0 | 0 | 0% |
| Jan 01/10 to May 31/11 | 1512 | 73 (4.8%) | 518 (34.3%) | 39.1% |  | 903 (59.7%) |  | 11 (0.7%) | 7 (0.4%) | 1.1% |
| **MAG-CP, by epoch (N)** | **3143** | **1162** | **947** |  |  | **742** |  | **213** | **79** |  |
| Jun 01/11 to Sep 30/11 | 270 | 44 (16.3%) | 81 (30.0%) | 46.3% |  | 134 (49.6%) |  | 8 (3.0%) | 3 (1.1%) | 4.1% |
| Oct 01/11 to Mar 31/12 | 406 | 121 (29.8%) | 111 (27.3%) | 57.1% |  | 149 (36.6%) |  | 23 (5.7%) | 2 (0.5%) | 10.2% |
| Apr 01/12 to Sep 30/12 | 380 | 120 (31.5%) | 118 (31.1%) | 62.6% |  | 99 (26.0%) |  | 29 (7.6%) | 14 (3.7%) | 11.3% |
| Oct 01/12 to Mar 31/13 | 379 | 159 (41.8%) | 118 (31.1%) | 73.1% |  | 67 (17.6%) |  | 22 (5.8%) | 13 (3.4%) | 9.2% |
| Apr 01/13 to Sep 30/13 | 418 | 166 (39.7%) | 131 (31.3%) | 71.1% |  | 77 (18.5%) |  | 33 (7.9%) | 11 (2.6%) | 10.6% |
| Oct 01/13 to Mar 31/14 | 383 | 181 (47.3%) | 99 (25.8%) | 73.1% |  | 66 (17.2%) |  | 28 (7.3%) | 9 (2.3%) | 9.7% |
| Apr 01/14 to Sep 30/14 | 346 | 128 (37.0%) | 118 (34.1%) | 71.1% |  | 58 (16.8%) |  | 28 (8.1%) | 14 (4.0%) | 12.1% |
| Oct 01/14 to Mar 31/15 | 310 | 140 (45.2%) | 94 (30.3%) | 75.5% |  | 47 (15.2%) |  | 24 (7.7%) | 5 (1.6%) | 9.4% |
| Apr 01/15 to Sep 30/15 | 251 | 103 (41.0%) | 77 (30.7%) | 71.7% |  | 45 (17.9%) |  | 18 (7.2%) | 8 (3.2%) | 10.4% |

** Optimal use refers to both women who received MgSO4 for fetal NP when indicated, as well as women who did not receive MgSO4 for fetal NP when it was not indicated. Underuse refers to eligible women who should have received MgSO4 for fetal NP but did not. Suboptimal use refers to women who received MgSO4 too early (not within 24hr before birth) or at ≥32 weeks.*

**Table S5. Overall odds ratios for use of MgSO4 for fetal NP as derived from segmented regression analysis of the nine centres that contributed data to both pre-MAG-CP and MAG-CP eras***

|  | **Optimal use** | **p-value** | **Underuse** | **p-value** | **Suboptimal use** | **p-value** |
| --- | --- | --- | --- | --- | --- | --- |
| Odds ratio for use in pre-MAG-CP, per month | 1.005 [0.997, 1.01] | 0.195 | 0.99 [0.99, 1.00] | 0.084 | 1.15 [1.08, 1.22] | <0.001 |
| Immediate change in odds just after intervention | 1.75 [1.45, 2.12] | <0.001 | 0.49 [0.35, 0.70] | <0.001 | 2.32 [1.07, 5.05] | 0.034 |
| Change in odds ratio after intervention compared to pre-MAG-CP, per month | 1.02 [1.00, 1.04] | 0.070 | 0.97 [0.95, 0.99] | 0.004 | 0.88 [0.83, 0.94] | <0.001 |
| Odds ratio for use in MAG-CP era, per month | 1.02 [1.01, 1.04] | <0.001 | 0.96 [0.95, 0.98] | <0.001 | 1.01 [1.00, 1.03] | 0.048 |

**Adjusted for antenatal administration of corticosteroids*

**Table S6: Sensitivity analyses of overall utilization rates of MgSO4 using data from the nine centres that contributed data to both pre-MAG-CP and MAG-CP eras**

|  | **N** | **Optimal use†** | | | **Underuse** | **Suboptimal use†** | | |
| --- | --- | --- | --- | --- | --- | --- | --- | --- |
|  |  | **Use when indicated** | **No use when not indicated** |  | **No use when indicated (missed)** | **Use that was too early** | **Use when not indicated** |  |
| **Pre MAG-CP, by epoch (N)** | **4025** | **66** | **1476** | **%** | **2466** | **10** | **7** | **%** |
| Aug 01/05 to Dec 31/08 | 1684 | 0 | 641 (38.1%) | 38.1 | 1043 (61.9%) | 0 | 0 | 0 |
| Jan 01/09 to Dec 31/09 | 990 | 0 | 359 (36.2%) | 36.3 | 631 (63.7%) | 0 | 0 | 0.1 |
| Jan 01/10 to May 31/11 | 1351 | 66 | 476 (35.0%) | 40.1 | 792 (58.6%) | 10 (0.7%) | 7 (0.5%) | 1.1 |
| **MAG-CP, by epoch (N)** | **3041** | **1111** | **915** | **%** | **725** | **211** | **79** | **%** |
| Jun 01/11 to Sep 30/11 | 261 | 38 (14.6%) | 80 (30.7%) | 45.2 | 132 (50.6%) | 8 (3.1%) | 3 (1.1%) | 4.2 |
| Oct 01/11 to Mar 31/12 | 393 | 117 (29.8%) | 104 (26.5%) | 56.2 | 147 (37.4%) | 23 (5.9%) | 2 (0.5%) | 6.4 |
| Apr 01/12 to Sep 30/12 | 365 | 114 (31.2%) | 116 (31.8%) | 63.0 | 92 (25.2%) | 29 (13.1%) | 14 (3.8%) | 11.8 |
| Oct 01/12 to Mar 31/13 | 367 | 153 (41.7%) | 114 (31.1%) | 72.8 | 65 (17.7%) | 22 (6.0%) | 13 (3.5%) | 9.5 |
| Apr 01/13 to Sep 30/13 | 411 | 162 (39.4%) | 130 (31.6%) | 71.0 | 76 (18.5%) | 32 (7.8%) | 11 (2.7%) | 10.5 |
| Oct 01/13 to Mar 31/14 | 375 | 177 (47.2%) | 95 (25.3%) | 72.5 | 66 (17.6%) | 28 (7.5%) | 9 (2.4%) | 9.9 |
| Apr 01/14 to Sep 30/14 | 330 | 118 (35.8%) | 112 (33.9%) | 69.7 | 58 (17.6%) | 28 (8.5%) | 14 (4.2%) | 12.7 |
| Oct 01/14 to Mar 31/15 | 296 | 133 (44.9%) | 89 (30.1%) | 75.0 | 46 (15.5%) | 23 (7.8%) | 5 (1.7%) | 9.5 |
| Apr 01/15 to Sep 30/15 | 243 | 99 (40.7%) | 75 (30.9%) | 71.6 | 43 (17.7%) | 18 (7.4%) | 8 (3.3%) | 10.7 |

|  | **Overall**  **(N=11)** | **Highly engaged (N=7)** | **Less engaged**  **(N=4)** |
| --- | --- | --- | --- |
| **KT Local team** |  |  |  |
| N members* | 9 [7, 15] | 9 [8,15] | 9 [4, 14] |
| KT Local Team Meetings | 0 [0,1] | 0 [0,4] | 0 [0,0] |
| **Barriers & Facilitators forms completed** |  |  |  |
| N times* | 3 [2,4] | 4 [3,4] | 2 [2,2] |
| N respondents (mean)* | 15 [13,18] | 18 [15, 19] | 13 [10, 16] |
| **Central to local activities** |  |  |  |
| N teleconferences participated* | 6 [4,9] | 8 [6,15] | 2 [2,3] |
| Site Visit* | 8 (72.7%)† | 6 (85.7%) | 2 (50%) |
| **Local activities** |  |  |  |
| Local grand rounds | 8 (72.7%) | 6 (85.7%) | 2 (50%) |
| In-services/Education Sessions | 8 (72.7%) | 6 (85.7%) | 2 (50%) |
| Informal one-on-one discussions | 4 (36.4%) | 3 (42.9%) | 1 (25%) |
| **Use of tools** |  |  |  |
| e-Learning module* | 10 (90.9%) | 7 (100%) | 3 (75%) |
| Protocol for MgSO4 for fetal neuroprotection* | 11 (100%) | 7 (100%) | 4 (100%) |
| Early implementation of local protocol | 6 (54.5%) | 3 (42.9%) | 3 (75%) |
| Pre-printed physician orders | 4 (36.4%) | 3 (42.9%) | 1 (25%) |
| Presentation materials (one/more) | 1 (9.1%) | 1 (14.3%) | 0 |
| Handouts (one/more) | 8 (72.7%) | 6 (85.7%) | 2 (50%) |
| Use of reminders (one/more) | 7 (63.6%) | 7 (100%) | 0 |
| **Data collection & participation** |  |  |  |
| Early buy-in for MAG-CP* | 6 (54.5%) | 4 (57.1%) | 2 (50%) |

**Table S7. Determinants of engagement of participating sites in MAG-CP**

**These components were directly measured by central team. The remainder were self-reported measures.*

*†Although eight site visits were conducted, these covered ten sites, as two smaller sites joined the nearest large centre during our visit.*

**Table S8. Components of engagement and relation to optimal use.**

|  | **High optimal use (N=6)** | **Low optimal use (N=5)** | **p-value** |
| --- | --- | --- | --- |
| **Overall engagement score** |  |  |  |
| Highly engaged | 3 (50%) | 4 (80%) | 0.500 |
| Less engaged | 3 (50%) | 1 (20%) |  |
| **KT Local team** |  |  |  |
| N members | 8 [6, 15] | 11 [9, 14] | 0.272 |
| KT Local Team Meetings | 0 [0,4] | 0 [0,0] | 0.562 |
| **Barriers & Facilitators forms completed** |  |  |  |
| N times | 3 [2,4] | 3 [2,4] | 0.925 |
| N respondents (mean) | 15 [10,16] | 19 [17,21] | 0.355 |
| **Central to local activities** |  |  |  |
| N teleconferences participated | 6 [4,9] | 6 [4,10] | 0.854 |
| Site Visit | 6 (100%) | 3 (60%) | 0.182 |
| **Local activities** |  |  |  |
| Local grand rounds | 3 (50%) | 4 (80%) | 0.546 |
| In-services/Education Sessions | 3 (50%) | 4 (80%) | 0.546 |
| Informal one-on-one discussions | 2 (33.3%) | 2 (40%) | 0.999 |
| **Use of tools** |  |  |  |
| e-Learning module | 6 (100%) | 4 (80%) | 0.455 |
| Protocol for MgSO4 for fetal neuroprotection | 6 (100%) | 5 (100%) | 0.999 |
| Early implementation of local protocol | 5 (83.3%) | 2 (40%) | 0.242 |
| Pre-printed physician orders | 2 (33.3%) | 1 (20%) | 0.999 |
| Presentation materials (one/more) | 0 | 1 (20%) | 0.455 |
| Handouts (one/more) | 4 (66.7%) | 3 (60%) | 0.999 |
| Use of reminders (one/more) | 3 (50%) | 4 (80%) | 0.546 |
| **Data collection & participation** |  |  |  |
| Early buy-in for MAG-CP | 5 (83.3%) | 1 (20%) | 0.08 |

**Table S9. Antenatal MgSO4 use at GA 24-31^+6^ weeks by indication, from Jan 1/11 to Sep 30/15.**

| **Time period (for infant date of birth)** | **N** | **Antenatal MgSO4 for any indication** | **For fetal neuroprotection** | **For maternal hypertension †** |
| --- | --- | --- | --- | --- |
| **Pre-MAG-CP** | **1179** | **232** | **31** | **0** |
| Jan 1/11 to May 31/11 | 1179 | 232 (19.7%) | 31/232 (13.4%) | 0/232 |
| **MAG-CP** | **12929** | **6638** | **5283** | **102** |
| Jun 1/11 to Mar 31/12 | 2347 | 797 (34.0%) | 492/797 (61.7%) | 0/797 |
| Apr 1/12 to Mar 31/13 | 3046 | 1411 (46.3%) | 1119/1411 (79.3%) | 1/1411 (0.1%) |
| Apr 1/13 to Mar 31/14 | 3053 | 1664 (54.5%) | 1362/1664 (81.9%) | 5/1664 (0.3%) |
| Apr 1/14 to Mar 31/15 | 2994 | 1837 (61.4%) | 1529/1837 (83.2%) | 38/1837 (2.1%) |
| Apr 1/15 to Sep 30/15 | 1489 | 929 (62.4%) | 781/929 (84.1%) | 58/929 (6.2%) |
| p-value 1 (trend: 1-6)* |  | <0.0001 | NA• | <0.0001 |
| p-value 2 (trend: 3-6)* |  | <0.0001 | 0.001 | <0.0001 |

Notes: **antenatal MgSO4 could have been received at any point before delivery, even remotely before delivery during a prior admission to hospital.

*P-value computed for trend over time using GEE model for period 1-6 and period 3-6 that represents the term of MAG-CP KT interventions.

• GEE model could not converge due to a site having very few numbers in the earlier time periods.

† NOTE that CNN does not contain information about the CPN indicator conditions of preterm labour, preterm prelabour rupture of membranes (PPROM) (only rupture of membranes that was artificial or spontaneous), short cervix, prolapsed membranes, antepartum haemorrhage, or intrauterine fetal growth restriction diagnosed before birth
